# Neuroimmune dynamics and brain aging: mechanisms and consequences

**DOI:** 10.3389/fnagi.2025.1715045

**Published:** 2025-11-19

**Authors:** Ludmila Müller, Svetlana Di Benedetto, Viktor Müller

**Affiliations:** Max Planck Institute for Human Development, Center for Lifespan Psychology, Berlin, Germany

**Keywords:** aging brain, neuroinflammation, immunosenescence, glial cells, neurons, immune cells, multilayered networks, neurodegenerative diseases

## Abstract

Brain aging is accompanied by profound changes in neuroimmune interactions that shape the balance between resilience and vulnerability. Under healthy conditions, glial cells, neurons, vascular elements, and peripheral immune inputs cooperate to sustain homeostasis. With advancing age, however, immune remodeling and systemic inflammaging drive shifts in microglial surveillance, astrocytic reactivity, and neuronal susceptibility, creating conditions that compromise synaptic function and cognitive performance. These processes unfold along a continuum, from subtle impairments in normal aging to maladaptive dynamics that accelerate neurodegenerative disease. Sex differences, epigenetic regulation, and systemic influences—including the gut microbiome, metabolic state, and lifestyle factors—further modulate these trajectories. Here, we synthesize current knowledge on the cellular, systemic, and molecular mechanisms that govern neuroimmune aging, emphasizing how their dysregulation contributes to cognitive decline and disease vulnerability. We also highlight emerging conceptual frameworks, such as multilayer network modeling and resilience biomarkers, that provide a foundation for integrative approaches to brain aging. Understanding these interconnected systems underscores the necessity of viewing brain aging not solely through a CNS-centric lens, but as a networked process influenced by distal organs, circulating immune cells, microbial communities, and lifestyle factors—setting the stage for integrative models of neuroimmune dynamics in aging. Clarifying how these dynamic interactions unfold and what their consequences are is essential for developing strategies to preserve cognitive health and mitigate the burden of neurodegeneration in an aging society.

## 1 Introduction

Aging is increasingly understood not simply as a gradual decline in cellular and physiological function, but as a dynamic process in which systemic, neural, and immune systems interact continuously. The brain, once thought largely immune-privileged, is now recognized to be in constant dialog with the immune system. These neuroimmune interactions influence neuronal function, synaptic plasticity, and ultimately cognition (Liston et al., 2022; Müller et al., 2025b). With aging, this delicate balance tends toward dysregulation, and contributes both to regulative functional decline and heightened vulnerability to neurodegenerative diseases (Ransohoff, 2016; Di Benedetto et al., 2017).

One prominent hallmark of aging is inflammaging (Franceschi and Campisi, 2014), a chronic, low-grade inflammatory state that develops systemically and affects the central nervous system (CNS). Alongside immunosenescence, which refers to age-associated remodeling or decline in immune function, inflammaging creates a milieu characterized by increased numbers of pro-inflammatory cytokines, altered immune cell profiles, and elevated oxidative stress (Murdaca et al., 2022; Bauer et al., 2024).

In the aging brain, several structural and functional changes accompany this shift. For example, microglia—the brain’s resident innate immune cells—become “primed”: they show elevated expression of antigen-presentation markers, pattern recognition receptors, and pro-inflammatory cytokines, and are more reactive to peripheral insults (Norden and Godbout, 2013; Müller and Di Benedetto, 2024a, 2024b). Astrocytes also change from homeostatic to reactive states, losing supportive functions related to synaptic maintenance and metabolic regulation, while reinforcing inflammatory signaling. Neuronal populations accumulate DNA damage and experience impaired repair and clearance mechanisms, further contributing to neuroinflammation (Zhang et al., 2024; Müller and Di Benedetto, 2025b; Müller et al., 2025b).

Moreover, aging impacts the physical and barrier components of CNS-immune communication. Blood–brain barrier (BBB) integrity declines with age, tight junctions loosen, pericyte coverage decreases, and trans-endothelial transport shifts from specific (receptor-mediated) to more permissive, non-specific mechanisms. These changes facilitate greater infiltration of peripheral immune molecules or cells, which can exacerbate neuroimmune responses (Knox et al., 2022; Tamatta et al., 2025).

Crucially, systemic influences—including metabolic health, gut microbiota composition, lifestyle factors (e.g., diet, exercise, stress), and peripheral immune status—play an increasingly recognized role in modulating neuroimmune aging. Dysbiosis in the gut, for instance, can trigger low-grade peripheral inflammation and increase circulating pathogen- or damage-associated molecular patterns (PAMPs or DAMPs), which, via compromised barriers and immune crosstalk, propagate to the CNS (Yu et al., 2022; Müller and Di Benedetto, 2025a).

Together, these changes in cellular phenotypes, barrier properties, and systemic signals coalesce to produce a neuroimmune environment in the aged brain that is more susceptible to maladaptive responses. The consequences of this dysregulated neuroimmune dynamics include synaptic dysfunction, reduced neuroplasticity, cognitive decline, and an increased risk of neurodegenerative pathology such as Alzheimer’s disease, Parkinson’s disease, and others (Gaikwad et al., 2024).

In this mini review, we aim to provide a comprehensive overview of neuroimmune dynamics in brain aging, with a focus on the mechanisms by which immune, glial, neuronal, vascular, and systemic processes interact over time. We highlight how these interactions shape both adaptive and maladaptive trajectories of aging, and how their consequences span from subtle functional decline to increased vulnerability to neurodegenerative diseases. By integrating insights across molecular, cellular, and systemic levels, we seek to frame brain aging as a multilayer network process, offering a conceptual foundation for future interdisciplinary research in neuroimmunology and aging.

## 2 Baseline neuroimmune networks in the healthy brain: a brief overview

**Figure 1:**
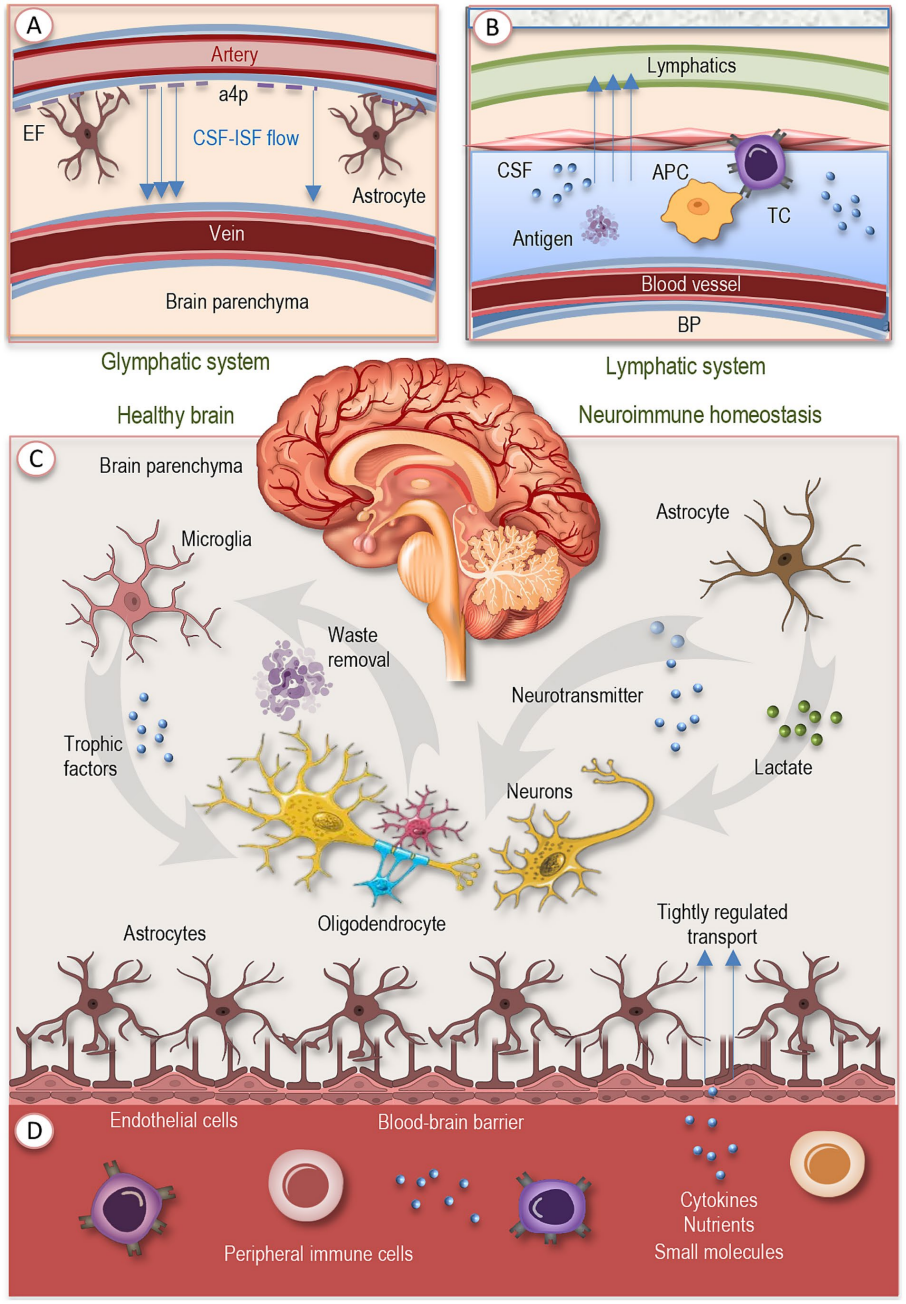
Baseline neuroimmune networks in the healthy brain. Neuroimmune homeostasis arises from the coordinated action of cellular agents, vascular interfaces, and fluid drainage systems. **(A)** The glymphatic pathway drives CSF circulation through periarterial and perivenous routes, aided by aquaporin-4–polarized astrocytic endfeet, and supports metabolic waste clearance. **(B)** Meningeal lymphatic vessels drain CSF and meningeal interstitial fluid to peripheral lymph nodes, enabling antigen presentation without parenchymal inflammation. **(C)** Microglia, long-lived myeloid cells, constantly survey the local environment, prune synapses, clear debris, and release trophic factors. Astrocytes complement these functions by maintaining ion balance, neurotransmitter turnover, metabolic support, and synaptic regulation. **(D)** Endothelial cells, supported by pericytes and astrocytic endfeet, form the blood–brain barrier, which restricts leakage, regulates molecular transport, and preserves barrier integrity. Together, these integrated layers provide surveillance, waste removal, metabolic support, and tightly regulated immune signaling, ensuring a resilient neuroimmune equilibrium under healthy conditions. EF, endfeed; a4p, aquaporin-4-polarized astrocytic endfeet; CSF, cerebrospinal fluid; IF, interstitial fluid; BP, brain parenchyma; APC, antigen-presenting cell; TC, T cell.

In youth and adulthood, neuroimmune homeostasis depends on the coordinated activity of drainage pathways, selective barriers, structural interfaces, and specialized glial cells, which together regulate molecular exchange, clear metabolic waste, and maintain immune surveillance (Figure 1). This interconnected architecture ensures that the CNS remains both responsive and protected, balancing the demands of function and defense. Before exploring this network at the cellular level, it is first essential to outline the principal drainage routes and the selective barriers that shape the CNS’s internal milieu, providing the framework upon which cellular interactions are orchestrated.

A crucial component of CNS homeostasis is the network of fluid drainage and antigen-clearance pathways. The glymphatic system (Figure 1A) ensures convective flow of cerebrospinal fluid (CSF) through arterial perivascular spaces, interstitial space, and eventually venous perivascular paths, aided by aquaporin-4 polarization in astrocyte endfeet (Silva et al., 2021; Müller and Di Benedetto, 2025a). This pathway supports removal of metabolic waste and maintenance of extracellular milieu. Imaging studies show that its function (for example assessed via DTI-ALPS, the diffusion tensor imaging along perivascular space index) declines with advancing adult age, especially after midlife, and that lower glymphatic efficacy correlates with declines in cognition and structural changes like thinning of key memory-related cortical zones (Li et al., 2022; Wang et al., 2023; Müller and Di Benedetto, 2025a).

In addition, meningeal lymphatic vessels provide routes by which soluble molecules and antigens from CSF and meningeal interstitial fluid are drained to peripheral lymph nodes, enabling antigen presentation and immune surveillance outside the brain parenchyma (Figure 1B). At steady state, immune cell populations in the dura and leptomeninges interact with these drainage pathways, capturing CNS-derived antigens and contributing to peripheral immune awareness of CNS status with minimal inflammatory disturbance (Louveau et al., 2018; Chachaj et al., 2023).

At the cellular level, microglia act as central orchestrators of CNS homeostasis. These long-lived, self-renewing myeloid cells adopt ramified morphologies in the steady state, continuously survey their environment, prune redundant synapses, clear debris, and release trophic factors that sustain neuronal health and network integrity (Figure 1C). Disruption of their signature homeostatic transcriptional profile leads rapidly to altered morphology, loss of molecular identity, and disturbances in neurogenesis and synaptic integrity (Kierdorf and Prinz, 2017; Müller and Di Benedetto, 2025b; Müller et al., 2025b).

Astrocytes operate in parallel with microglia: in healthy brains they contribute to ion homeostasis, regulation of neurotransmitter turnover, support of metabolic-needs (e.g., provision of lactate), and reinforcement of synaptic function (Figure 1C). Structural complexity of astrocytes (especially in larger mammalian brains) allows them to interact with many thousands of synapses, shaping and modulating neural circuit function even in resting conditions (Lopez-Ortiz and Eyo, 2024; Müller et al., 2025b).

Endothelial and other vascular cells form selective barriers, most prominently the BBB, that mediate molecular and cellular traffic between the circulation and neural tissue (Figure 1D). This barrier is built by tightly connected endothelial cells supported by astrocytic endfeet and pericytes, which together maintain its integrity and selective permeability. Under homeostatic conditions they restrict leakage, tightly regulate transport of nutrients, cytokines, and small molecules, and maintain barrier integrity. Meanwhile, peripheral immune cells and molecules (cytokines, growth factors) are largely excluded from the parenchyma but can sense and respond to cues at barrier interfaces, meningeal spaces, and perivascular zones, providing a form of immune surveillance without extensive infiltration. Microglia, astrocytes, endothelial cells, oligodendrocyte precursor populations, and neurons are all parts of this surveillance and signaling ensemble (Kabba et al., 2018; Müller et al., 2025b).

Together, this architecture supports a baseline of neuroimmune equilibrium: each cellular type performs dual roles in maintenance and surveillance, the barriers limit accidental activation, and drainage systems ensure waste and antigen manageability. These integrated layers set the stage for how perturbations such as aging, disease, systemic stress propagate across scales. As organisms grow older, however, systemic immune remodeling reshapes the inputs to this network, gradually altering the conditions under which neuroimmune communication takes place.

## 3 Systemic aging and immune remodeling

**Figure 2:**
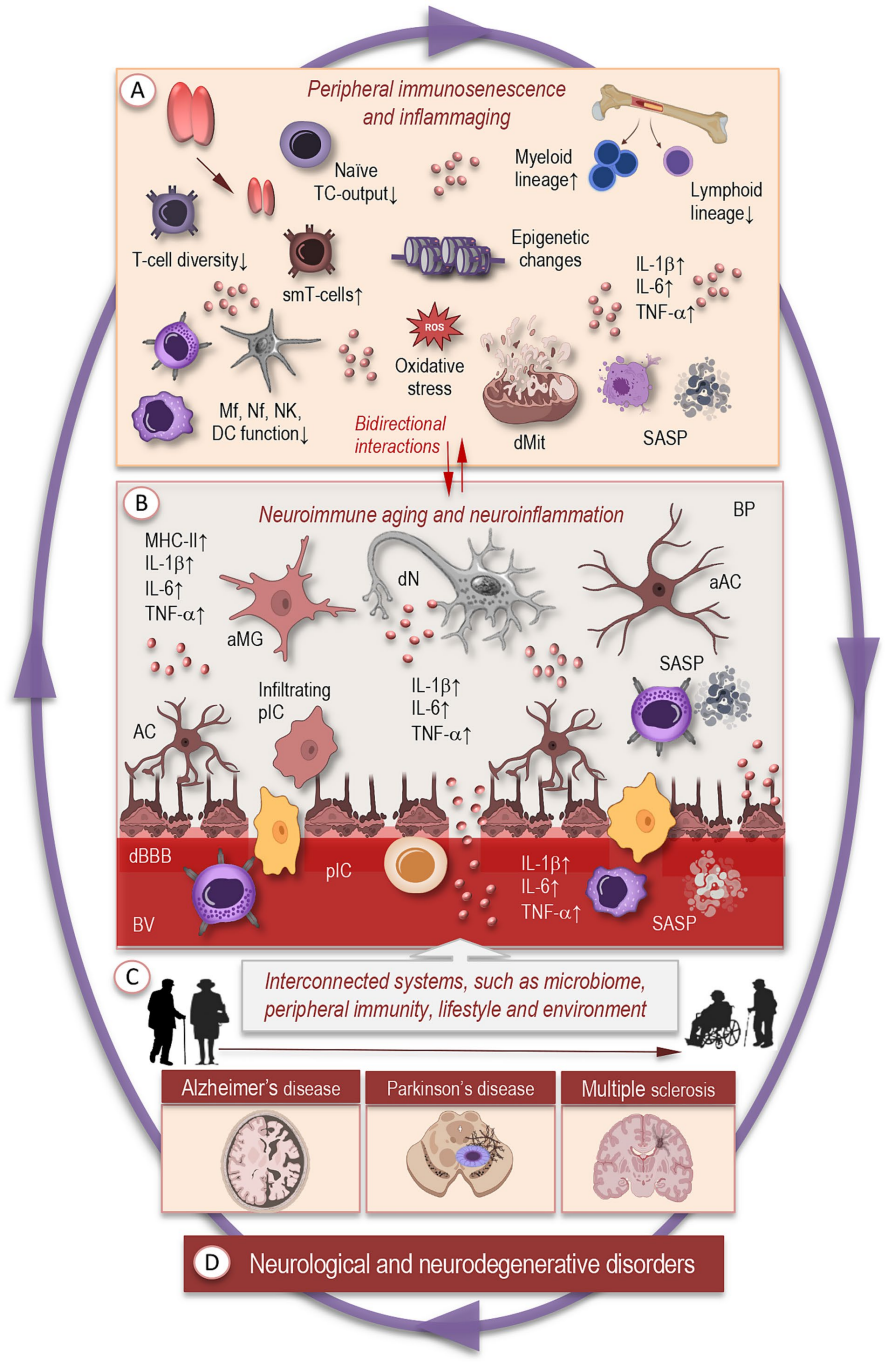
Systemic and neuroimmune remodeling with aging. **(A)** Aging triggers widespread changes in the immune system, including thymic involution, reduced production of naïve T cells, accumulation of memory and senescent T-cell clones, and skewed hematopoiesis favoring myeloid over lymphoid lineages. Innate immune cells—macrophages, neutrophils, natural killer cells, and dendritic cells—exhibit elevated basal activation but blunted responses to acute challenges. Cellular features such as mitochondrial dysfunction, impaired autophagy, metabolic dysregulation, and DNA damage accumulate over time, promoting inflammaging and reduced immune surveillance. **(B)** In the brain, these systemic changes contribute to altered neuroimmune states, reflecting bidirectional interactions between aging peripheral and central immune systems that reciprocally shape inflammatory balance. Microglia adopt primed or dystrophic phenotypes with elevated basal cytokine expression, impaired phagocytosis, and retracted or fragmented processes. Astrocytes display metabolic dysregulation, reduced synaptic support, and increased reactive states, while neurons experience energy deficits, synaptic dysfunction, and heightened vulnerability to maladaptive glial signaling. Dysregulated communication among these cell types amplifies inflammatory and stress signals, linking normal aging to early stages of neurodegenerative vulnerability. **(C)** Interconnected systems beyond the brain —including systemic immune signals, microbial metabolites, and lifestyle influences—modulate brain immune states and further shape neuroimmune aging. **(D)** Chronic dysregulation of these networks contributes to vulnerability and progression toward neurodegenerative diseases, such as Alzheimer’s disease, Parkinson’s disease, and multiple sclerosis. TC, T cells; IL, interleukin; TNF, tumor necrosis factor; smT, senescent memory T cells; Mf, macrophage; Nf, neutrophil; NK, natural killer; DC, dendritic cells; dMT, dysfunctional mitochondria; SASP, senescence-associated secretory phonotype; aMG, activated microglia; AC, astrocyte; aAC, activated astrocyte; dN, degenerating neuron; BV, blood vessel; pIC, peripheral immune cells; cBBB, compromised blood–brain-barrier; BP, brain parenchyma.

Aging triggers widespread changes in the immune system—collectively termed immunosenescence—which do not simply reduce function, but reorganize immune architecture and regulation in a dynamic, remodeling process (Pawelec, 2012). Alongside immunosenescence, there is often a parallel increase in systemic, low-grade inflammation that emerges even in the absence of overt infection (Figure 2A). These two phenomena are intertwined: immunosenescence diminishes immune surveillance, while inflammaging can be driven by senescent somatic cells and dysregulated immune activation (Fulop et al., 2017; Ajoolabady et al., 2024; Goyani et al., 2024).

One of the earliest and most striking contributors to immunosenescence is thymic involution. Over time, the thymus loses mass and becomes replaced by adipose tissue; its capacity to produce naïve T cells and to support central tolerance diminishes, reducing the diversity of the T-cell receptor repertoire and increasing the proportion of memory and senescent T-cell clones. This reduces adaptive immune responsiveness to novel antigens (Pawelec, 2012; Palmer, 2013; Müller and Pawelec, 2015; Müller et al., 2019a).

At the level of innate immunity, aging is associated with decline in functions such as phagocytosis, pathogen detection, and pathogen killing. Macrophages, neutrophils, natural killer cells, and dendritic cells show altered responsiveness; often there is an elevated basal activation state, but a blunted response to acute challenges. Reactive oxygen species (ROS) production, mitochondrial dysfunction, impaired autophagy, and metabolic dysregulation are recurrent cellular features associated with these changes (Goyani et al., 2024).

Another layer of remodeling is found in hematopoietic stem and progenitor cells: their output becomes skewed, with a relative increase in myeloid vs. lymphoid lineages (Figure 2A). This bias results in more proinflammatory myeloid cells (monocytes, neutrophils) and fewer naïve lymphocytes. Telomere shortening, epigenetic drift, and accumulation of DNA damage in progenitor cells contribute to these shifts (Wang et al., 2011; Wells et al., 2024).

At the molecular level, immune remodeling involves changes in signaling pathways, epigenetic regulation, post-translational modifications, and the secretory phenotypes of senescent cells. For example, senescence-associated secretory phenotype (SASP) factors such as IL-6, TNF-*α*, and other inflammatory cytokines increase over time; concurrently, regulators like cytokine inducible SH2-containing protein (CISH) have been implicated in reducing lysosomal function in aging T cells, which contributes to mitochondrial DNA release and persistent inflammation (Wang S, et al., 2025).

Gut microbiota alterations also form part of systemic immune remodeling. Aging is associated with changes in microbial diversity, increased permeability of the gut barrier, and greater translocation of microbial products (PAMPs) into circulation, which can chronically stimulate innate immune compartments and feed into inflammaging. Nutritional status and dietary components (e.g., micronutrients, immunonutrients) modulate these effects, affecting levels of systemic inflammatory markers such as C-reactive protein, IL-6, and TNF-α (Yu et al., 2022; Müller and Di Benedetto, 2025a).

These systemic changes have several consequences for the brain: reduced clearance of peripheral insults, altered immune signaling, elevated basal inflammation that compromises CNS barriers, and the priming of glial cells. Together, they create a milieu in which neuroimmune communication becomes increasingly fragile and prone to maladaptation. As a result, the cellular constituents of the brain’s immune network—particularly microglia, astrocytes, and neurons—operate under altered baseline conditions. The next section examines how these shifts manifest at the cellular level, tracing the progression from adaptive responses in youth to maladaptive phenotypes that drive neuroimmune aging.

## 4 Cellular mechanisms of neuroimmune aging

With aging, cellular components of the brain immune environment progressively shift in identity, function, and interaction. These changes in microglia, astrocytes, and neurons, together with altered glia–neuron communication, form key mechanisms underlying neuroimmune aging (Figure 2B).

Microglia, in their youthful state, continuously monitor the brain environment, rapidly clearing debris, pruning synapses, and responding subtly to shifts in extracellular signals. With aging, however, microglia adopt a sensitized or senescent phenotype: they display elevated baseline expression of pro-inflammatory cytokines, antigen presentation molecules (e.g., MHC II), and pattern recognition receptors, even in the absence of acute insult. Morphologically, these aged microglia often show dystrophic features, including deramified or fragmented processes, spherical soma, and reduced motility (Norden and Godbout, 2013; Norden et al., 2015; Kierdorf and Prinz, 2017; Carr et al., 2025; Müller and Di Benedetto, 2025b; Müller et al., 2025b). Such changes are accompanied by impaired phagocytosis (for example, of amyloid in Alzheimer’s disease models), decreased ability to resolve inflammation, and a reduced capacity to respond adaptively to metabolic or oxidative stress (Heneka et al., 2015; Edler et al., 2021; Heneka et al., 2024).

Single-cell transcriptomic profiling of the human orbitofrontal cortex across the adult lifespan shows early deviation of microglial gene programs in late adulthood, particularly in pathways related to lipid metabolism and protein homeostasis (Frohlich et al., 2024). Complementary studies reveal age- and APOE-dependent immunometabolic perturbations in human microglia, implicating lipid handling and metabolic dysregulation in the transition toward disease-associated phenotypes (Patel et al., 2022).

Astrocytes also undergo profound aging-related alterations: their metabolic profile changes, synaptic support declines, and reactive states become more common (Figure 2B). In adult aging brains, astrocytes show a switch from aerobic glycolysis to oxidative phosphorylation, which reduces lactate supply to neurons—this metabolic rebalancing may heighten neuronal energetic deficits since neurons concurrently downregulate expression of Krebs cycle and related metabolic genes (e.g., mdh1, mdh2; Patel et al., 2022; Gildea and Liddelow, 2025).

Additionally, aging astrocytes show mitochondrial fragmentation, reduced mitochondrial biogenesis, and lowered resilience to oxidative stress. On the synaptic side, aging is associated with downregulation of synaptogenic factors (e.g., thrombospondin family genes), and upregulation of negative regulators of synapse formation, which contributes to reduced dendritic spine density and weakening of synaptic plasticity (Gildea and Liddelow, 2025). Spatial transcriptomic studies in aging mice reveal widespread astrocytic reprogramming, including increased expression of inflammatory and complement genes, particularly in white matter tracts where astrocytes interact with degenerating myelin and activated microglia (Wang L, et al., 2025).

Neurons themselves are not passive in this landscape; they both suffer from, and contribute to, neuroimmune aging (Acevedo et al., 2023; Müller and Di Benedetto, 2025b; Müller et al., 2025b, 2025c). Neuronal energy deficits (via reduced metabolic enzyme expression and declines in mitochondrial efficiency) reduce neurons’ ability to maintain ionic gradients, synaptic transmission, and repair. These deficits render neurons more vulnerable to damage by maladaptive glial responses, such as excessive release of pro-inflammatory cytokines (IL-1β, TNF-*α*), reactive oxygen species, or complement activation. Additionally, the dysregulation of supporting trophic factors or metabolic intermediates from astrocytes and microglia can tip the balance toward synapse loss, dendritic retraction, and eventual functional decline (Acevedo et al., 2023).

Single-nucleus profiling of human orbitofrontal cortex demonstrates that aging neurons, particularly inhibitory subtypes, accumulate transcriptomic changes overlapping with those seen in neurodegenerative and psychiatric conditions, involving synaptic transmission, RNA splicing, and protein homeostasis (Frohlich et al., 2024). These deficits are further amplified when glial support wanes and inflammatory signals intensify.

Crosstalk among these cellular compartments becomes progressively dysregulated. In healthy conditions, neuron-derived signals such as CX3CL1 and CD200 restrain glial reactivity, but their expression declines with aging, weakening these inhibitory checkpoints. Distress signals from metabolically challenged neurons (e.g., DAMPs) increasingly activate microglia and astrocytes, while senescent cells scattered through cortical and hippocampal regions release pro-inflammatory factors that propagate local activation foci in the aged mouse brain (Haidar et al., 2022; Kiss et al., 2022; Müller and Di Benedetto, 2025b). Spatial transcriptomics highlight that such inflammatory hotspots frequently align with white matter tracts, where microglia, astrocytes, and degenerating oligodendrocytes converge, reinforcing regional vulnerability (Wang L, et al., 2025).

Altogether, aging shifts the cellular neuroimmune landscape from resilient surveillance to a state of heightened sensitivity, metabolic fragility, and maladaptive amplification of stress signals. Microglia become primed, astrocytes reactive, neurons vulnerable, and their communication increasingly feeds back into cycles of inflammation and dysfunction. These changes create a cellular framework that bridges normal aging processes with the earliest stages of neurodegenerative vulnerability. Understanding the timing, cell-type specificity, and molecular underpinnings of these shifts is essential to map how early neuroimmune dysregulation may presage later cognitive decline and disease susceptibility.

## 5 Sex differences in neuroimmune aging

Sex profoundly shapes the trajectory of neuroimmune aging, influencing both systemic immune remodeling and the cellular dynamics of the brain. These differences arise from hormonal regulation, chromosomal factors, and lifelong variations in immune system activity, and they contribute to divergent susceptibilities to age-related cognitive decline and neurodegeneration (Ruigrok et al., 2014; McCarthy et al., 2017; Müller et al., 2025c). Alzheimer’s disease (AD) shows higher prevalence and faster cognitive decline in female, while Parkinson’s disease (PD) occurs more frequently in male and manifests with sex-specific symptom profiles. Multiple sclerosis (MS) is diagnosed more often in female, yet male typically experience a more aggressive and neurodegenerative disease course (Ribbons et al., 2015; Laws et al., 2018; Gilli et al., 2020; Philipe de Souza Ferreira et al., 2022; Boccalini et al., 2025; Cattaneo and Pagonabarraga, 2025).

Hormonal regulation is a central driver of sex-specific neuroimmune trajectories. Estrogens, progesterone, and androgens modulate microglial activation, astrocytic reactivity, and neuronal resilience. Estrogens generally exert anti-inflammatory and neuroprotective effects by dampening pro-inflammatory cytokine release, enhancing mitochondrial function, and promoting synaptic plasticity (Maggioli et al., 2016; Zarate et al., 2017). In contrast, declining estrogen levels during menopause are associated with heightened neuroinflammation and reduced glial support, particularly in hippocampal and cortical circuits involved in memory. Androgens, though less studied, appear to modulate microglial activity and protect against excessive synaptic pruning, with declining testosterone in aging males linked to increased vulnerability to glial-driven inflammation (Maggio et al., 2005; Dengri et al., 2025; Müller et al., 2025c).

Beyond endocrine regulation, immune aging follows distinct trajectories in males and females. Female generally exhibit stronger baseline immune responses, with greater adaptive immune activity but also higher risk of autoimmune conditions. With aging, this heightened immune tone interacts with declining sex hormones to amplify pro-inflammatory signaling in the brain, accelerating the shift of microglia and astrocytes toward reactive phenotypes (Klein and Flanagan, 2016; Di Benedetto et al., 2019a). In male, immune aging tends to follow a slower trajectory with less pronounced inflammaging, but often coupled with earlier vascular dysfunction and metabolic decline that indirectly affect neuroimmune homeostasis (Giefing-Kroll et al., 2015; Müller et al., 2025c).

Sex differences in neuroinflammation reflect not only hormonal influences but also genetic and epigenetic factors. The X chromosome, enriched in immune-related genes, contributes to stronger immune activation in females through partial escape from X-chromosome inactivation, enhancing surveillance but also increasing risk of overactivation and autoimmunity. These mechanisms shape glial function and long-term immune programming, underpinning sex-specific vulnerability and resilience in aging and disease (Lopez-Otin et al., 2023; Feng et al., 2024).

Sex-specific epigenetic regulation, including DNA methylation, histone modifications, and microRNAs, programs long-term immune and glial phenotypes (McCarthy et al., 2017; Chinn et al., 2021). In aging brains, females generally maintain more stable methylation, whereas males show greater variability at immune loci, indicating divergent epigenetic control of neuroinflammation (Yusipov et al., 2020; Lopez-Otin et al., 2023; Shirokova et al., 2023). Transcriptomic studies reveal stronger inflammatory reprogramming in female hippocampal microglia, with upregulation of complement genes and glycolytic metabolism characteristic of the DAM phenotype, while aged males display a weaker response (Ramamurthy et al., 2022; Kang et al., 2024; Singh and Paramanik, 2024).

Mouse studies indicate that male microglia adopt pro-inflammatory states early in life, whereas female microglia show greater phagocytic capacity and injury responsiveness in adulthood (Villa et al., 2018). In humans, transcriptomic profiling has identified female-enriched disease-associated microglia (FDAMic), more abundant in female with late-onset AD and correlated with disease severity (Wu et al., 2024). These cells display activation and antigen-presentation signatures but reduced phagocytic function, likely linked to impaired estrogen receptor signaling in APOE4 carriers.

In the aging mouse hippocampus, female microglia exhibit stronger upregulation of senescence-associated and inflammatory pathways than males, together with greater expression of age-dependent genes and epigenetic modifications (Ocañas et al., 2023). Li et al. found that female microglia progress through aging phases more gradually, whereas male microglia transition more abruptly toward aged phenotypes (Li et al., 2023).

In aged rodents, alterations in microglial function correlate with sex differences in cognitive decline. Ince et al. (2023) reported that male aged rats display stronger inflammatory priming of microglia that associates with worse performance in memory tasks (Ince et al., 2023). Such findings underscore that sex not only influences cellular immune phenotypes but also translates into measurable differences in cognitive outcomes.

These converging lines of evidence point to sex as a critical modifier of neuroimmune aging, shaping vulnerability to disease and resilience to stress. Building on this cellular and molecular framework, the next section turns to systemic modulators that interact with brain immune networks to further influence aging trajectories.

## 6 Interconnected systems beyond the brain

The aging brain operates within a complex network influenced by peripheral systems, metabolites, and lifestyle factors (Figure 2C). Increasing evidence highlights the role of the gut–brain–immune axis as a central modulator of neuroimmune health. The intestinal microbiota produces metabolites, neurotransmitter precursors, and short-chain fatty acids that shape microglial maturation, astrocytic function, and BBB integrity. With aging, microbial diversity declines and pro-inflammatory taxa expand, amplifying systemic cytokine levels and skewing microglia toward reactive states—a process that contributes to the inflammaging (Pluta et al., 2020; Müller and Di Benedetto, 2025a; O'Riordan et al., 2025).

For example, in aged mice, transplantation of gut microbiota from old donors worsens the neurological outcome after ischemic stroke, mediated in part by elevated valeric acid in the bloodstream and increased IL-17 signaling. Young recipients of old microbiota show heightened inflammation and poorer recovery, illustrating that microbial metabolites can prime peripheral immunity and exacerbate central injury (Zeng et al., 2023). Another recent study shows that gut microbial communities in aged mice possess increased immunogenic potential: aged microbiota more strongly activate Toll-like receptor 4 signaling, correlate with elevated circulating levels of lipopolysaccharide-binding protein, and induce heightened systemic inflammation when transplanted into young germ-free mice. These findings directly connect age-related shifts in microbiota to increased peripheral immune activation and barrier compromise (Caetano-Silva et al., 2024).

Peripheral immune cells and circulating factors further interact with the brain’s immune milieu. T cells, monocytes, and systemic cytokines can access the CNS through the choroid plexus, meningeal lymphatics, or transient BBB permeability. With aging, T-cell populations shift toward pro-inflammatory phenotypes, monocyte recruitment patterns change, and baseline levels of cytokines such as IL-6, TNF-*α*, and IFN-*γ* rise, all of which influence microglial priming, astrocytic reactivity, and neuronal susceptibility (Yang et al., 2020; Müller and Di Benedetto, 2025b). These systemic inputs help explain why peripheral health—immune competence, infection history, and chronic inflammation—can modulate brain aging trajectories.

Recent work in AD mouse models shows that rejuvenation of peripheral immune compartments (e.g., via young bone marrow transplantation) can restore some Aβ clearance, reduce systemic inflammatory cytokines, improve cell–cell communication among immune cells, and ameliorate cognitive deficits. This highlights that peripheral immunosenescence is not just a bystander but contributes causally to central neuroinflammation and disease pathology (Sun et al., 2024).

Lifestyle and environmental factors provide additional layers of modulation in aged population. Diets rich in fiber, polyphenols, and omega-3 fatty acids preserve microbiome diversity, maintain intestinal barrier integrity, and reduce pro-inflammatory signaling in macrophages and other immune cells. Conversely, high-fat or pro-oxidant diets accelerate gut barrier breakdown, systemic endotoxin influx, and microglial activation. In aged humans, regular physical activity attenuates microglial priming and preserves synaptic function, while chronic stress, sleep disruption, and sedentary behavior accelerate glial reactivity, impair metabolic homeostasis, amplify peripheral inflammatory signaling, and degrade BBB function (Phillips, 2017; Müller and Di Benedetto, 2025a).

Together, these interconnected systems—microbial communities, peripheral immunity, lifestyle and environment—create a multilayered network that shapes neuroimmune aging (Figure 2C). The extent to which each node (gut, systemic immune cells, and lifestyle) influences the brain depends on factors such as age, sex, genetic background, and cumulative environmental exposure. Understanding how these upstream systems interact with cellular and molecular mechanisms in the brain is essential for mapping the trajectories from adaptation to maladaptation—and for identifying possible intervention points to sustain cognitive resilience.

## 7 Consequences of dysregulated neuroimmune dynamics

Neuroimmune processes that support plasticity and repair in early life can become sources of dysfunction when compensatory mechanisms erode, such that continued or excessive immune activity crosses adaptive thresholds and accelerates tissue damage. Longitudinal imaging and experimental work *in vivo* and vitro indicate that once microglial priming, astrocytic dysfunction, or barrier compromise pass critical limits, vulnerability to acute insults and chronic decline increases markedly (Wyss-Coray and Mucke, 2002; Malpetti et al., 2023; Wang et al., 2023; Xue et al., 2024).

In typical aging, cognitive change most often reflects a slow accumulation of subtle neuroimmune alterations—mild increases in baseline cytokine tone, faint astrocytic reactivity, reduced synaptic plasticity—that translate into declines in processing speed, attention, and episodic memory yet remain compatible with independent function. In a subset of individuals these same processes intensify and enter pathological trajectories: sustained inflammation, persistent glial activation, and progressive synaptic loss drive a transition from mild functional impairment to clinically meaningful cognitive decline. Plasma and CSF biomarkers follow this continuum: longitudinal increases in phosphorylated tau species and neurofilament light (NfL) identify individuals progressing from preclinical states to symptomatic disease (Bilgel et al., 2023).

Neurodegenerative disorders exemplify the end points of prolonged neuroimmune dysregulation (Figure 2D). In AD, for example, in vivo measures of glial activation (TSPO PET) co-localize with amyloid and associate with subsequent atrophy and cognitive deterioration, suggesting that chronically activated innate immunity both reflects and contributes to disease progression. Similarly, elevated systemic inflammatory markers and specific cytokine profiles correlate with accelerated cognitive decline across human cohorts, linking peripheral inflammaging with central vulnerability (Rossano et al., 2024).

In PD, neuroimmune dysregulation is increasingly recognized as a central contributor to disease onset and progression. Microglia in the substantia nigra display chronic activation, releasing pro-inflammatory cytokines and reactive oxygen species that exacerbate dopaminergic neuron vulnerability (Kwon and Koh, 2020; Gao et al., 2023; Müller et al., 2025a). Postmortem and in vivo studies reveal infiltration of peripheral T cells into affected brain regions, suggesting that adaptive immunity participates alongside innate immune responses in shaping neurodegeneration (Brochard et al., 2009; Sommer et al., 2017). Longitudinal cohort studies further show that elevated systemic inflammatory markers, such as C-reactive protein and IL-6, correlate with faster motor and cognitive decline in PD patients (Scalzo et al., 2010; Kim et al., 2022). Thus, these findings highlight PD as a exemplary case where central and peripheral immune alterations converge to accelerate selective neuronal loss, underscoring that neuroimmune dysregulation is not only a consequence but also a driver of disease pathology.

Alzheimer’s and Parkinson’s disease illustrate two distinct yet converging routes by which maladaptive neuroimmune responses accelerate pathology—whether through impaired clearance of protein aggregates or heightened vulnerability of specific neuronal populations. Similar immune–glial imbalances occur across other neurodegenerative diseases, suggesting that neuroimmune dysregulation is a unifying mechanism in late-life brain disorders. Together, these observations support a model in which dysregulated neuroimmune dynamics unfold along a continuum from resilience to vulnerability: many age-related changes remain contained for years, but when buffering capacity is lost the system tips toward degeneration. Mapping the biomarkers and imaging signatures that mark these tipping points offers a pragmatic route to detect transitions from adaptive aging to emerging pathology.

## 8 Perspectives and future directions

The recognition that neuroimmune dysregulation underlies both normal cognitive decline and diverse neurodegenerative diseases calls for a shift from reductionist views of single cell types or isolated pathways toward integrative, network-based frameworks. Aging of the brain is not solely a matter of neuronal attrition but reflects multilayered interactions among glia, neurons, peripheral immune cells, systemic mediators, and environmental inputs.

Analyzing complex biological systems such as the immune system and CNS is challenging due to their multidimensionality, context dependence, and nonlinear regulation (Müller et al., 2019b). Traditional single-layer network models have revealed sex- and CMV-related differences in interactions between inflammatory biomarkers, hormones, immune cells, and cognitive outcomes (Di Benedetto et al., 2019b), but they cannot fully capture cross-domain dependencies. Multilayer network (MLN) approaches extend this framework by integrating interactions across molecular, cellular, and tissue levels, enabling simultaneous analysis of intra- and inter-layer dynamics. In neuroimmune contexts, nodes can represent cell types while inter-layer links reflect cytokines, neurotransmitters, or receptor–ligand signaling, with mass spectrometry and multiplex assays providing the quantitative basis (Müller et al., 2025c). Applied to neuroinflammation, MLNs can reveal how glial reactivity or cytokine perturbations propagate through the system, altering neuronal function and resilience.

Recent single-cell and multimodal studies are already beginning to map out these multilayered networks. For example, Sun et al. survey how cellular clocks, epigenetic drift, altered cell–cell interactions, and molecular signaling across cell types contribute to aging-related decline and identify candidate rejuvenation targets (Sun et al., 2025). In another work, Jones et al. employ multilayer network analysis to show how physical activity, synaptic peptide integrity, and phosphorylated tau interact to shape both pathology and cognition in Alzheimer’s disease (Jones et al., 2024).

MLN analyses show that genes associated with the same disease cluster within distinct “disease modules” of molecular interaction networks, supporting the disease module hypothesis (Menche et al., 2015; Sharma et al., 2015; Buphamalai et al., 2021). Comparable modular organization has been observed in immune and cognitive networks, where biomarkers and performance measures form tightly interconnected modules, reflecting functional interdependence (Di Benedetto et al., 2019b). Likewise, bacteria–metabolite MLN analyses revealed that specific metabolic pathways are closely linked to altered microbial communities (Duran et al., 2021). Large-scale proteomic studies further demonstrate that immune cells communicate through complex, highly coordinated signaling networks—akin to social systems—offering crucial insights into the molecular architecture of disease-related communication (Rieckmann et al., 2017).

To move forward, three key conceptual directions are particularly important. First, resilience biomarkers: molecular signatures that stratify individuals by how far they are from tipping points. The recent study DNA methylation signature of a lifestyle-based resilience index for cognitive health identifies methylation loci correlated with lifestyle, cognitive reserve, and slower decline (Zhang et al., 2025). Similarly, cell-type abundance measures in AD reveal that higher glial (especially astrocyte and oligodendrocyte) proportions correlate with cognitive resilience even in the presence of high pathology (O'Neill et al., 2024).

Second, combining imaging, fluid biomarkers, and network topology can reveal early signs of adaptation failure. For instance, studies of functional brain networks demonstrate that features such as k-core resilience are associated with preserved episodic memory and processing speed in middle-aged and older adults, even in the presence of age-related changes (Stanford et al., 2022). Also, CSF biomarkers reflecting glial reactivity show strong associations with BBB leakage and white matter lesions, offering mechanistic links between peripheral/homeostatic disruption and central pathology (Dai et al., 2024).

Third, precision stratification that accounts for sex, genetic risk (e.g., APOE genotype), lifestyle, and systemic health. Longitudinal cohort work demonstrates that healthy lifestyle factors—including diet, physical activity, cognitive engagement—attenuate genetic risk of cognitive decline in APOE-ε4 carriers (Xu et al., 2024). Moreover, multicomponent lifestyle scores predict cognitive function proximate to death even after controlling for neuropathologies, suggesting modifiable factors shape late-life resilience (Dhana et al., 2024).

In moving toward interventions, combining mechanistic experimental models with human longitudinal data will be crucial in determining when resilience is lost and how to restore it. Advances in multi-omics, spatial mapping of cell neighborhoods in aging brain tissue, and dynamic network modeling of biomarkers are promising tools for this challenge. If the field succeeds in defining reliable early markers of maladaptive neuroimmune shifts and tailoring stratified prevention strategies, the goal of preserving cognitive health into advanced age becomes more attainable.

## 9 Conclusion

Neuroimmune interactions are central to the trajectory of brain aging, shaping the balance between resilience and vulnerability. While subtle shifts in glial function and immune signaling underpin normal cognitive changes, maladaptive dynamics drive the transition toward neurodegenerative disease. Sex differences, systemic modulators, and environmental factors add further complexity, emphasizing the need for integrative, network-based perspectives. By combining molecular profiling, longitudinal studies, and systems modeling, future research can illuminate the tipping points at which protective responses become pathogenic. Such insights will be crucial for developing strategies aimed not only at treating disease but at sustaining healthy cognitive aging across the lifespan.
